# Does City Public Service Distance Increase Sense of Gain to Public Health Service? Evidence from 1394 Migrant Workers in Six Provinces

**DOI:** 10.3390/ijerph19106131

**Published:** 2022-05-18

**Authors:** Minghui Fu, Chuanjiang Liu, Yuting Ma, Liukun Wang

**Affiliations:** 1 School of Insurance, Guangdong University of Finance, Guangzhou 510521, China; mhfu@gduf.edu.cn; 2 College of Economics and Management, Nanjing University of Aeronautics and Astronautics, Nanjing 211106, China; chjliu@nuaa.edu.cn; 3 College of Economics and Management, Nanjing Forestry University, Nanjing 210037, China; 4 College of Economics & Management, Huazhong Agricultural University, Wuhan 430070, China; liukunwang@webmail.hzau.edu.cn

**Keywords:** accessibility, public health service, migrant worker, sense of gain, generalized propensity score matching

## Abstract

Increasing the well-being of migrant workers is one of the key objectives of promoting equality and safe, people-oriented, and sustainable social development, as well as inclusive globalization. With the equalization reform of the public health system and the reduction of frictions between cities, the well-being of the sense of gain to public health service (SGPHS) of migrant workers has attracted widespread attention. Based on the migrant worker thematic survey data in 2017 and the city statistical data in six destination cities, this study constructed and measured the sense of gain to public health service index and city public service distance index, and then studied the effects of city public service distance on the SGPHS of migrant workers and the heterogeneous effect. The results showed that the SGPHS of Chinese migrant workers is at a moderate level and presents spatial differences. Under the dual mechanism of preference reinforcement effect and public service discount effect, the effect of city public service distance on the SGPHS of migrant works shows an inverted U-shaped relationship, and the results of the endogeneity test by the generalized propensity score matching model are robust. The city public service distance has a significant non-linear effect on the public health service accessibility and provision for migrant workers, as well as on second-generation, low-income migrant workers, and migrant workers in central and western regions. The results provide beneficial insights for the formulation of rational public service policies.

## 1. Introduction

Improving well-being is the ultimate goal to be pursued by human society [1,2,3]. With about one billion people moving globally, the well-being of migrants has received widespread attention from social and academic fields, because migrants are among the most vulnerable groups in their destination areas owing to their low socio-economic background and exclusion from the local public service system [4,5,6]. Much of the economic literature has devoted attention to the subjective well-being or happiness of international migrants in developed countries [7,8,9], such as in the United States [9], the United Kingdom [10], and Britain and Canada [11], while the specific well-being of migrant workers in developing countries has been less understood. As a typical developing country, China has experienced an exponential increase in the number of migrant workers. With the equalization reform of the public health system and the reduction of frictions in rural-urban migration, the effects of this equalization reform have attracted the attention of policymakers [12]. In this study, we focus on the construction and measurement of the specific subjective well-being of Chinese migrant workers—the sense of gain to public health service (SGPHS)—and explain the differences in migrant workers’ SGPHS from the perspective of the city public service distance.

The SGPHS is the objective acquisition and subjective evaluation of the provision and accessibility of public health services. It is a sense of well-being that encompasses both material gain and spiritual satisfaction. The SGPHS of migrant workers measures individual well-being in terms of public health services and relates to sustainable economic and social development [13]. Lower SGPHS may trigger a strong sense of deprivation among migrant workers, which directly influences their physical and mental health and even augments social instability (e.g., crimes) [14,15]. Given the importance of the well-being of migrant workers, policies to promote the equalization of public health services and social integration are required. Therefore, a comprehensive explanation of the effect of each factor on the SGPHS of migrant workers from the perspective of individual-level, household-level, city-level, and inter-city level is a significant concern to the Chinese government and policymakers that warrants much research attention. In recent years, the sense of gain has been regarded as one of the most appropriate indicators to measure human well-being, as it measures individuals’ objective gain and subjective evaluation of economic and social development, social justice, and governance [16]. It has received increasing attention from scholars and policymakers [17]. The Chinese government proposes to ensure that all people have a greater sense of gain in the process of building and sharing development, which is in line with the vision for the post-2015 United Nations (UN) development agenda of achieving inclusive, people-centered, and sustainable global development [18]. In welfare economics, the sense of gain is similar to happiness and subjective life quality. However, happiness tends to be more psychological and subjective, and the “Easterling paradox” questions the appropriateness of happiness as a measure of reform effectiveness [13]. In contrast, the sense of gain emphasizes a sense of tangible gain, focuses on the welfare of vulnerable groups, and is thus more inclusive.

Recently, a nascent strand of literature has studied the sense of gain [19]. For example, the overall sense of gain of Chinese people is moderate and increases with rise in social status [20]. Existing studies mainly focus on the following three aspects: (1) the conceptualization of sense of gain, for example, employee sense of gain is defined as the subjective feeling of obtaining various objective benefits due to employees’ efforts at work [17]; the subjective sense of gain is defined as people’s positive psychological experience and subjective satisfaction with the achievements of China’s reform and opening up [21]. (2) The measurement of sense of gain, for example, the employee sense of gain is measured by two dimensions: employee’s material sense of gain and employee’s spiritual sense of gain with a 14-item ESG scale [17]; the overall sense of gain is measured by self-assessment of life satisfaction, and the relative sense of gain is measured by self-assessment of social status and communication confidence [21]. (3) The influencing factors on sense of gain by empirical test, for example, community identity, played a mediating role between socioeconomic status and sense of gain [15], and along with subjective socioeconomic status has a positive statistical correction effect on sense of gain from health-care reform [16].

The SGPHS in our study is of great significance for policymakers to realize inclusive public health, because it reflects the effectiveness of the implementation of governance and the equalization of public health services [22]. However, research on the measurement of the SGPHS is still in its infancy, because of the lack of first-hand survey data or better quantitative indexes. Besides, most of the literature studies the effects of individual-level, family-level, and city-level factors on the sense of gain of migrant workers, while there are few studies on the effect of inter-city differences on the SGPHS of migrant workers [20]. This study aims to fill this gap.

Based on 1394 observations from the 2017 migrant worker thematic survey and statistical data from 195 cities, this study first constructed an index system for the SGPHS of migrant workers in terms of public health service accessibility and provision and measured the SGPHS with the entropy weight method. Second, the influencing factors on migrant workers’ SGPHS were discussed from four aspects: individual-level, household-level, city-level, and inter-city level, mainly focusing on the effects of city public service distance on the SGPHS of migrant workers. Third, the heterogeneity effects of migrant workers’ SGPHS were compared between groups defined by generation, income, and region. Finally, the endogeneity of city public service distance and SGPHS was tested by generalized propensity score (GPS) matching. The results showed that the SGPHS of Chinese migrant workers is at a moderate level and presents spatial differences; under the dual mechanisms of the “preference reinforcement effect” and “public service discount effect”, the effect of city public service distance on the SGPHS of migrant works shows an inverted U-shaped relationship, and the results of the endogeneity test by the GPS matching model are robust; city public service distance has a significant non-linear effect on public health service accessibility and provision of migrant workers, as well as second-generation, low-income migrant workers, and migrant workers in central and western regions.

The rest of the paper is organized as follows: Section 2 provides a conceptual framework for the relationship between city public service distance and the SGPHS of migrant workers; Section 3 introduces the data, variables, and methods utilized; Section 4 shows the measurement results of the SGPHS of migrant workers, descriptive statistical analysis, estimation results of the effect of city public service on the SGPHS of migrant workers, the results of heterogeneity analysis, and the results of GPS matching; Section 5 provides the discussion; and Section 6 offers conclusions and policy implications.

## 2. Theoretical Analysis and Research Hypotheses

Inspired by the literature on cultural distance in cultural economics [23,24], this study defines city public service distance as the difference in public service supply between the destination cities and the hometown of migrant workers, and then investigates the association between city public service distance and the SGPHS of migrant workers. The impact of city public service distance on the SGPHS of migrant workers is driven by the preference reinforcement effect and public service discount effect. The preference reinforcement effect refers to migrant workers preferring public services that are similar to those in their hometowns, e.g., education, health, and medical care, because of their familiarity with these public services in their hometown. The public service discount means that the public service in the destination cities is not well recognized or accepted by migrant workers because of the differences in cultural and social backgrounds between the hometown and destination cities, bringing about a decrease in the value of public service. In other words, this discount is similar to the depreciation of resources owing to inefficient usage of public service [23,25].

The marginal effect of city public service distance is positive when the preference reinforcement effect is bigger than the public service discount effect. In this case, the magnitude of accessibility and provision of public health services is similar between the migrant workers’ hometown and destination city, and the city public service distance increases their SGPHS.

**Figure 1:**
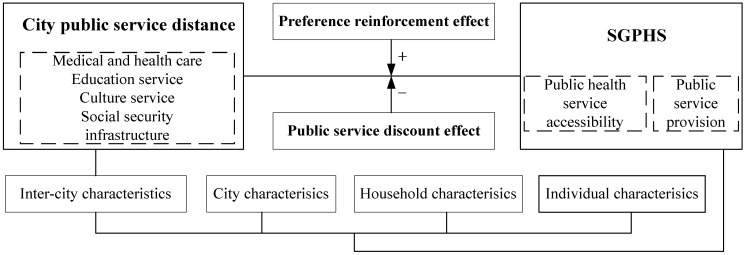
Core research framework.

Besides, the marginal effect of city public service distance is negative when the preference reinforcement effect is smaller than the public service discount effect. In this case, there are large gaps in the magnitude of accessibility and provision of public services between migrant workers’ hometowns and destination cities, and then the public service in the destination city is not recognized or accepted by migrant workers. As a result, higher city public service distance will reduce the SGPHS of migrant workers and bring about a loss of SGPHS. Specifically, when the SGPHS reaches the critical level, the marginal effect of city public service distance decreases, and the increase in the city public service distance will no longer be consistently effective in enhancing well-being. Thus, migrant workers in regions with higher city public service distances fall into a larger happiness gap. As we explain from the perspective of the social comparison theory [26,27], migrant workers in destination cities develop a negative psychology of exploitation, i.e., relative deprivation, when they find it more difficult to utilize public services in their destination cities than urban residents, which reduces the SGPHS of migrant workers. The core research framework is shown in Figure 1.

Based on the above theoretical analysis, two hypotheses that the city public service distance affects the SGPHS of migrant workers are put forward.

**H1.**  *When the preference reinforcement effect is dominant, a larger city public service distance increases the preference of migrant workers for the destination city, and enhances the SGPHS of migrant workers.*

**H2.**  *When the public service discount effect is dominant, a larger city public service distance increases the sense of deprivation of migrant workers, which in turn reduces the SGPHS of migrant workers.*

## 3. Methodology and Data

### 3.1. Data Acquisition

#### 3.1.1. Migrant Worker Thematic Survey Data

The major data of this study include 1394 migrant workers from 195 hometowns and six destination cities, which are derived from the migrant worker thematic survey data in 2017 and the China City Statistics Yearbook. The migrant worker thematic data survey is conducted by the key research base of humanities and social science in the Ministry of Education of China—Center of Economic Development Research of Wuhan University. The survey focuses on the key issues in the process of citizenship of migrant workers, covering many important issues such as individuals and families, migration and integration, work and labor, health and land transfer, and agricultural production. The survey was conducted in the second half of 2017 in six representative cities of Beijing, Shanghai, Guangzhou, Zhengzhou, Wuhan, and Nanchang. Within each of the above survey cities, the sample was selected utilizing a combination of city and urban area quotas, sample points, and sample random sampling, in which the urban area quotas were proportional to the population size of the central areas of the survey cities, and the sample points and survey sample were randomly selected from the labor units and streets in these central urban areas with a high concentration of migrant workers. The responders were migrant workers without local urban *hukou* but who had lived in destination cities for more than six months. A total of 1500 questionnaires were completed for this survey, and 1394 valid questionnaires were obtained by removing the samples with missing observations.

In our sample, first, for industry, 30.96% of these migrant workers work in manufacturing industry, 16.14% work in the construction industry, 11.76% work in the wholesale and retail trade industry, 10.19% work in residential services repair and other services industries, 13.85% work in the accommodation and food service activities industry, and 6.46% work in the transportation and storage industry. Second, for employment status, 78.07% are employees, 6.69% are employers, 7.84% are self-employed, while 7.40% have other types of employment status, such as domestic helpers. Finally, those aged 30 and below accounted for 35.19%, those aged between 30 and 50 accounted for 56.55%, while those aged 50 and above accounted for 8.25%. The data of individual characteristics of the samples are roughly consistence with the 2017 migrant workers monitoring survey report [28].

#### 3.1.2. China City Statistical Yearbook

The China City Statistical Yearbook is an annual statistical publication that comprehensively reflects the economic and social development of cities in China. It was published by the Department of Urban Social and Economic Investigations of the National Bureau of Statistics in China. This study collects the data from the China City Statistics Yearbook, including 195 prefecture-level public service statistical indexes in 2017.

### 3.2. Variables Measurement

#### 3.2.1. Sense of Gain to Public Health Service (SGPHS) Index and Measurement

The dependent variable is the subjective well-being of migrant workers in terms of the provision and accessibility of urban public health services, i.e., the SGPHS. The previous research on public service-specific subjective well-being has mainly focused on the city public service equalization index or individual overall subjective well-being, as per Dustmann and Okatenko [29], Helliwell et al. [22], Li et al. [30], and Wu et al. [31]. However, there are few studies on the construction of specific subjective well-being indicators at the individual level, and even fewer studies on the construction of indicators combining public health service accessibility and public health service provision. As public health services are mainly provided to residents rather than the entire urban population, it is more effective and accurate to identify the SGPHS of migrant workers at the individual level than at the city level. In addition, the existing research is mostly focused on the provision of public health service, ignoring the accessibility of public health service. The accessibility of public health services directly determines the actual access of migrant workers to public health services as they are restricted by *hukou* and often excluded from the urban public health service system. Therefore, based on the classical theory of public health service and relevant studies, we incorporate public health service accessibility into the index system of SGPHS.

The index system of SGPHS of migrant workers includes two dimensions: (1) public health service accessibility, which measures the matching quality of the public health service supply system and demand system [32]. According to the public health access theory [33], we obtained the indicators of public health service accessibility including availability, approachability, accommodability, affordability, and acceptability. Rows 3–7 of Table 1 describe the five indicators of public service accessibility. (2) Public health service provision, which measures the evaluation of migrant workers on the quantity of public health service in the destination cities. A large body of literature has put forward different index systems from different perspectives to measure a city’s public health service provision [12,30]. After summarizing the classification criteria of public health service provision as well as the availability of data [30,34], we selected three public health service provision indicators: public health service, medical services, and community management service.

The main reasons for selecting these indicators are as follows: first, these three types of public health services are the most needed and commonly used for migrant workers, and the indicators of community management services include a wide variety of public services (e.g., infrastructure, welfare, and safety). Second, compared with other types of public health services, most of these public health services require financial support from the destination city governments [12,30], and migrant workers are most likely to be excluded from the utilization of these services. To sum up, we selected three indicators of public health service provision and five indicators of public service accessibility to construct the index system of SGPHS, as shown in Table 1.

The data of the migrant worker thematic survey in 2017 provide substantial items for this study to measure the SGPHS. In the dimension of public health service accessibility, for example, the indicator of availability is based on the respondent’s responses to the “availability score of public health service in the destination city” after describing the meaning of the indicator to them. In addition, for the dimension of public health service provision, the indicator of public health service is based on the respondent’s responses to “evaluation of the provision of public health service in the destination city”. For each response, respondents were asked to choose between “low”, “relatively low”, “average”, “relatively high”, and “high”, and assigned a score of 1–5 to each of the five options. The higher the score, the higher the respondent’s evaluation of the indicator. Finally, we obtain a dataset of eight indicators of the SGPHS of migrant workers, each of which is an ordinal variable with the value of (1, 5).

To ensure a high consistency among all indicators of the scale, this study conducts a reliability test by analyzing the Cronbach’s alpha of the scale and taking 0.70 as the standard for the internal consistency reliability [17]. The results show that the Cronbach’s alpha reliability coefficients of public service accessibility and public health service provision are 0.868 and 0.819, respectively (Table 1), indicating that the indicators of the two dimensions have relatively good reliability.

Based on the index system of SGPHS, this study adopts the deviation standardization method and entropy weight method to calculate the SGPHS of migrant workers. The entropy weight method is an objective weighting method that mainly relies on the discreteness of the data itself and can maximize the utilization of the information of each indicator. In general, given the different orientation effects of the positive and negative indicators, the numeric indicators need a pre-process to eliminate the interference influence of magnitude and positive and negative orientation [35]. In this study, the indicators are transformed into normalized values with a numerical range from 0 to 1 using a deviation standardization method (Formula (1)). For an indicator, the higher the normalized value, the higher the evaluation of the migrant workers of the SGPHS.

(1) $$X_{ij}=\left\{\begin{array}{l} \frac{a_{ij}-\min\{a_{1j},\ldots ,a_{nj}\}}{\max\{a_{1j},\ldots ,a_{nj}\}-\min\{a_{1j},\ldots ,a_{nj}\}}\left(positiveindicator\right)\\ \frac{m\mathrm{ax}\{a_{1j},\ldots ,a_{nj}\}-a_{ij}}{\max\{a_{1j},\ldots ,a_{nj}\}-\min\{a_{1j},\ldots ,a_{nj}\}}\left(negativeindicator\right) \end{array}\right.,i=1,\ldots ,n;j=1,\ldots ,m.,$$

where $X_{ij}$ refers to the normalized value for the jth (i=1,…,n;j=1,…,m) indicator of the SGPHS of the ith migrant worker ($X_{ij}\in [0,1]$). $a_{ij}$ is the jth numeric indicator of SGPHS. The $\max\{a_{1j},\ldots ,a_{nj}\}$ is the maximum value of the sample and the $\min\{a_{1j},\ldots ,a_{nj}\}$ is the minimum value of the sample.

After obtaining the normalized indicators of SGPHS, the weight of each indicator is further calculated by the entropy weight method. First, the initial evaluation matrix of SGPHS is obtained based on the standardized index. The proportion of the jth indicator value of the ith migrant worker in the sum of the jth indicator value of all the migrant workers is calculated as follows:

(2) $$P_{ij}\in \frac{X_{ij}}{\sum_{i=1}^{n}X_{ij}}\left(P_{ij}\neq 0\right),$$

where $P_{ij}$ is the proportion of the jth indicator of the ith migrant worker.

Second, the information entropy ($E_{j}$) of the jth indicator of SGPHS is calculated as follows:

(3) $$E_{j}=-k\sum_{i=1}^{n}P_{ij}\cdot \ln P_{ij},$$

where k=1/ln(n), k>0. The information entropy of each indicator is non-negative ($E_{j}\geq 0$) because the value of $P_{ij}$ is higher than 0 and lower than 1.

Third, the information entropy redundancy (discrepancy) of each indicator, and then the weight of that indicator in the sum of the information entropy redundancy of the whole indicator, is calculated, and thus the entropy weight of each indicator is obtained. The formula is as follows:

(4) $$W_{j}=\frac{1-E_{j}}{\sum_{j=1}^{m}\left(1-E_{j}\right)},$$

where $W_{j}$ is the entropy weight of the jth indicator, that is, the weight of the SGPHS.

The SGPHS of migrant workers is finally obtained by a weighted summation of the standardized values of each indicator and its entropy weight. The formula is as follows:

(5) $$SGPHS_{i}=\sum_{j=1}^{n}X_{ij}\cdot W_{j},$$

where $SGPHS_{i}$ is the SGPHS of the ith migrant worker. Thus, we can obtain the specific values of the SGPHS of migrant workers in this study.

#### 3.2.2. City Public Service Distance Index

The city public service distance is the key independent variable in this paper. Currently, the indicators measuring inter-city differences are mainly city scale, and public service provision, as per Chen et al. [36] and Li et al. [30]. However, to the best of our knowledge, there is little literature on measuring inter-city public service distance. In fact, according to the migration theory and cultural dimension theory [23,24], city public service distance has a significant impact on the subjective well-being of migrant workers. Therefore, by extending the theoretical framework of the cultural dimension to the study of public services [37,38,39], we constructed the city public service distance index to describe the overall public service proximity level between the destination city and hometown. The city public service distance (CPSD) is measured as follows:

(6) $$CPSD_{dh}=\frac{1}{n}\sum_{k=1}^{n}\frac{{\left(I_{kd}-I_{kh}\right)}^{2}}{V_{k}},k=1,2,\ldots ,n,$$

where $CPSD_{dh}$ is the public service distance between destination city d and hometown h, $I_{kd}$ and/and $I_{kh}$ refer to the kth public service indicator of destination city d and hometown h, respectively. $V_{k}$ is the variance of the kth public service indicator. 

We construct the city public service distance index from 15 indicators in five dimensions: medical and health care, education service, cultural service, social security, and infrastructure. These indicators in 195 cities were derived from the China City Statistical Yearbook. Table 2 shows the index system of city public service distance.

#### 3.2.3. Control Variables

We control a variety of variables to mitigate the possible spurious correlation as follows. The individual characteristics include age, age-squared, yearly income, and dummies for gender, education level, and marital status. The household characteristic comprises family size. The logarithm form of family size is taken. The city characteristics include eastern cities dummy and the average city wage, and the latter is taken in logarithm form. Besides, considering that there may be differences in the SGPHS of residents from different cities, we also control the inter-city characteristics by the city geodesic distance, which measures how geographical proximity affects the SGPHS of migrant workers. The city geodesic distance is calculated by utilizing the geo-mathematical model based on the geographic coordinates of the WGS (World Geodetic System) 1984.

### 3.3. Empirical Model

The effect of city public service distance on the SGPHS of migrant workers is proposed with the following nonlinear regression equation specification:

(7) $$SGPHS_{i}=\beta_{0}+\beta_{1}CPSD_{i}+\beta_{2}CPSD_{i}^{2}+\gamma Individual_{i}+\delta Family_{i}+\phi City_{i}+\theta Intercity+\mu_{i}$$

where the explained variable ($SGPHS_{i}$) is the sense of gain to public health service for migrant workers, which is a continuous variable and is calculated by two dimensions of public health service accessibility and public health service provision. The explanatory variable ($CPSD_{i}$) is the city public service distance of migrant workers, which is a continuous variable and is calculated by five dimensions of city public service distance between the destination city and hometown. The covariates include individual characteristics, household characteristics, city characteristics, and inter-city characteristics.

### 3.4. Statistical Analyses

We use Stata software version 16.0 to manage and analyse data. First, the deviation standardization method was utilized to eliminate the interference influence of magnitude and positive and negative orientation of each indicator, and the entropy weight method was adopted to calculate the SGPHS of migrant workers. Second, the destination-hometown difference index method was conducted to calculate the city public service distance index, and a geo-mathematical model was used to calculate the city geodesic distance index. Third, the nonlinear regression model was adopted to investigate the relationship between city public service distance and the SGPHS of migrant workers. Finally, generalized propensity score (GPS) matching was performed by using a bootstrapping approach based on 1000 bootstrap samples and a 95% confidence interval. to test the endogeneity between the city public service distance and SGPHS.

## 4. Results

### 4.1. Measurement Results of SGPHS

**Figure 2:**
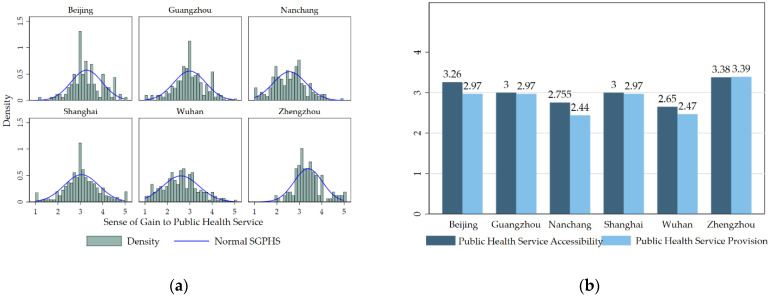
The sense of gain to public health service (SGPHS) of migrant workers from six cities in China. (**a**) Density distribution of SGPHS of migrant workers in cities; (**b**) average public health service accessibility and public health service provision of migrant workers in cities.

Figure 2 shows the distribution of the SGPHS of 1394 migrant workers in six destination cities. The density distribution in Figure 2a shows that the distribution of SGPHS of migrant workers in destination cities is close to the normal distribution, and the SGPHS is within the range of 2.5–3.5, i.e., the evaluation of SGPHS of migrant workers is moderate. Specifically, the SGPHS of migrant workers in eastern China is higher. Migrant workers in Zhengzhou, Beijing, Shanghai, and Guangzhou have the highest SGPHS, with values of 3.400, 3.276, 3.025, and 2.997, respectively, which are higher than the sample mean of 2.961. The SGPHS of migrant workers in Zhengzhou, Beijing, and Shanghai is higher than 3, indicating that the migrant workers in these cities have a higher SGPHS. Meanwhile, migrant workers in Nanchang and Wuhan have a lower SGPHS, with values of 2.579 and 2.607, respectively, reflecting the relatively low SGPHS of migrant workers in these cities.

Figure 2b shows the evaluation of migrant workers in six cities on public health service accessibility and provision. The public health service accessibility index for migrant workers in Beijing, Nanchang, and Wuhan is 3.26, 2.775, and 2.65, respectively, which is higher than their public health service provision index (2.97, 2.44, and 2.47, respectively). Although migrant workers in these three cities have access to public health services, the quality of public service available needs to be improved. In contrast, the index of public health service accessibility of migrant workers in Guangzhou, Shanghai, and Zhengzhou is 3.00, 3.00, and 3.38, respectively, which is comparable to that of public health service provision. Besides, the value of the public health service accessibility index and provision index of migrant workers in Zhengzhou is the highest, indicating the high social integration of migrant workers in Zhengzhou.

In summary, the well-being of migrant workers is affected by the city characteristics and by cultural differences between cities [40,41]. Differences in public services between destination cities and hometowns may result in different SGPHS in the destination cities. Therefore, this study will explain the difference in SGPHS of migrant workers from the perspective of public service distance between destination cities and hometowns.

### 4.2. Descriptive Analysis

Table 3 shows the descriptive statistical analysis of the variables employed in this study. The average SGPHS of migrant workers in cities is 2.96, indicating that their SGPHS is at a moderate level. Migrant workers’ evaluation of public health service accessibility is 3.03, and that of public health service provision is 2.89. The average public service distance is 5.92. Besides, 53% of the respondents in this study are male, the average age is 34 years old, 43% of the respondents have a junior middle school education, 28% of the respondents have a senior high school education, and 15% of the respondents have junior college and above. Furthermore, 57% of the sample is married, and the average annual income of migrant workers is 49,200 Yuan. The average family size of migrant workers is 4.57, and the average annual family income is 95,700 Yuan. The average geographic distance between destination cities and hometowns is 492 km.

### 4.3. Effects of City Public Service Distance on the SGPHS of Migrant Workers

The previous section shows the measurement results of the SGPHS of migrant workers and finds that there is spatial heterogeneity in the SGPHS of migrant workers. More importantly, what factors affect the SGPHS of migrant workers? However, relevant studies remain limited. We further study the effects of city public service distance on the SGPHS of migrant workers. According to the empirical model of SGPHS (Formula (7)), the effect of individual-level, household-level, city-level, and intra-city characteristics on SGPHS is studied (see Table 4). Model (1) presents the effects of city public service distance (CPSD) and CPSD-squared on SGPHS of migrant workers by the linear regression model. Model (2) adds controls for individual characteristics, model (3) adds controls for household characteristics, and model (4) adds controls for city characteristics. The parentheses of the model (1)–(4) are robust standard errors. The R-squared of all models is greater than 0.2, indicating that the regression results are quite robust.

The city public service distance has a significant effect on the SGPHS of migrant workers. Model (4) demonstrates an inverted U-shaped relationship between city public service distance and SGPHS of migrant workers, and the inflection point of this inverted U-shaped relationship is 2.18. The impact of city public service distance on the SGPHS of migrant workers is determined by the preference reinforcement effect and public service discount effect. The result reveals that, when the standardized index of city public service distance falls below 2.18, the preference reinforcement effect is bigger than the public service discount effect, thus the city public service distance has a positive effect on the SGPHS of migrant workers. When the standardized index of city public service distance exceeds 2.18, the preference reinforcement effect is smaller than the public service discount effect, thus the city public service distance negatively affects the SGPHS of migrant workers. In this study, city public service distance is mainly steadily increasing along with the SGPHS of migrant workers.

Individual characteristics significantly affect the SGPHS of migrant workers. Model (4) shows a U-shaped relationship between the age and SGPHS of migrant workers, and the inflection point of this U-shaped relationship is 35 years old. This indicates that, when the age of migrant workers reaches the threshold of 35 years old, their SGPHS increases with age. Migrant workers with the education of senior high school or junior college and above have a higher SGPHS than those with primary school education, which reflects the increased well-being brought about by higher levels of education. The coefficient of migrant workers’ income is negative and statistically insignificant. The result reveals that the “happiness paradox” indicates that the increase in income causes migrant workers’ expectation of public service to exceed utility, which in turn leads to a decrease in their SGPHS [42,43].

City characteristics have significantly increased the SGPHS for migrant workers. Migrant workers in eastern coastal cities have a higher SGPHS than those in municipalities. Specifically, migrant workers in eastern cities have 0.377 standard deviations higher SGPHS than those in central and western cities. The average local wage in model (4) does not significantly increase the SGPHS of migrant workers. Besides, the increase in the family size of migrant workers does not improve their SGPHS.

The city geodesic distance has increased the SGPHS of migrant workers; with every one standard deviation increase in the city geodesic distance, the SGPHS increases by 0.04 standard deviations. The result reveals that, although long-distance migrant workers incur higher social and psychological costs, their SGPHS is at least the same as that of short-distance migrant workers. However, the coefficients of city geodesic distance are insignificant. Besides, the effects of city public service distance on the SGPHS are higher than those of city geodesic distance. Thus, it is evident that city public service distance rather than city geodesic distance has a significant effect on the SGPHS of migrant workers.

### 4.4. Effects of City Public Service Distance on the Public Health Service Accessibility and Provision

Given that the SGPHS is composed of public health service accessibility and public health service provision, this subsection further studies the factors influencing both public health service accessibility and public health service provision. Table 5 reports the regression results of the determinants of public health service accessibility and public health service provision. Both model (5) and model (6) contain individual characteristics, household characteristics, city characteristics, and inter-city characteristics. 

Model (5) shows an inverted U-shaped relationship between city public service distance and public health service accessibility of migrant workers, and the inflection point of this inverted U-shaped relationship is 2.368. The result reveals that city public service distance has a positive effect on the public health service accessibility of migrant workers when the standardized index of city public service distance falls below 2.368, and a negative effect on the public health service accessibility of migrant workers when the standardized index of city public service distance rises above 2.368.

Model (6) shows an inverted U-shaped relationship between city public service distance and public health service provision of migrant workers, and the inflection point of this inverted U-shaped relationship is 2.111. The inflection point of city public service distance in model (5) is higher than that in the model (6), which may be due to the greater demand of migrant workers for public health service accessibility. Besides, the coefficients of city geodesic distance in model (5) and model (6) are positive but statistically insignificant.

### 4.5. Heterogeneity Analysis

**Figure 3:**
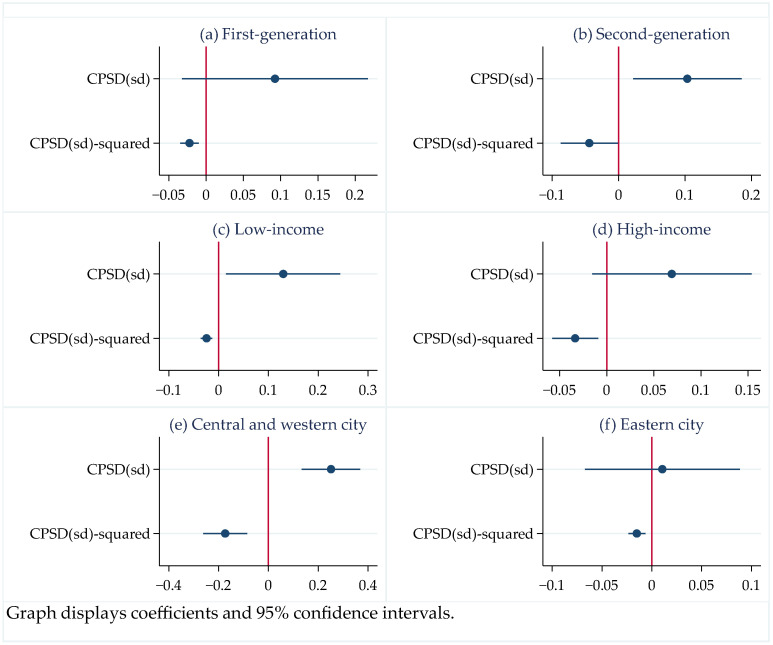
The heterogeneity effect of city public service distance on the SGPHS of migrant workers in terms of generations, income, and region. (**a**) The effect of city public service distance on the SGPHS of the first-generation migrant workers. (**b**) The effect of city public service distance on the SGPHS of the second-generation migrant workers. (**c**) The effect of city public service distance on the SGPHS of the low-income migrant workers. (**d**) The effect of city public service distance on the SGPHS of the high-income migrant workers. (**e**) The effect of city public service distance on the SGPHS of the migrant workers in central and western China. (**f**) The effect of city public service distance on the SGPHS of the migrant workers in eastern China.

Migrant workers have been divided into different subgroups with rapid economic development and continuous structural change over the past 40 years. However, it remains unclear whether there are any differences in the effects of factors on different subgroups. Answering this question may help to understand the equalization effect and be important for policy-makers in promoting the sustainable development of urbanization. We further explore the heterogeneous effects of each factor on different subgroups. In this subsection, we compare the heterogeneity effects of city public service distance on the SGPHS of migrant workers’ subgroups in terms of generation, income, and city type. Figure 3 shows the heterogeneity effects of city public service distance in terms of generation, income, and city type.

Migrant workers are divided into two groups according to whether their birth year is before 1980 or after 1980; that is, first-generation migrant workers and second-generation migrant workers, respectively. We analyze the effect of city public service distance on the SGPHS in these two subsamples of migrant workers.

Figure 3a,b demonstrate the heterogeneity effect of city public service distance on the SGPHS of the first-generation migrant workers and second-generation migrant workers. The results show that city public service distance has a significant nonlinear effect on the SGPHS of the second-generation migrant workers, but not on the SGPHS of the first-generation migrant workers. Figure 3b shows an inverted U-shaped relationship between city public service distance and SGPHS of second-generation migrant workers, and the inflection point of this inverted U-shaped relationship is 1.170. The result reveals that city public service distance has a positive effect on the SGPHS of second-generation migrant workers when the standardized index of city public service distance falls below 1.170, and a negative effect on the SGPHS of second-generation migrant workers when the standardized index of city public service distance rises above 1.170.

It is well known that there are significant differences in the socioeconomic status of high- and low-income groups. Is there any difference then in the SGPHS among migrant workers with different incomes? We further study the effect of the income heterogeneity of migrant workers. Migrant workers are divided into high-income and low-income groups according to whether their income is higher or lower than the mean. We analyze the effects of city public service distance on migrant workers’ SGPHS of different subgroups in separate regressions.

Figure 3c,d demonstrate the heterogeneity effect of city public service distance on the SGPHS of low-income migrant workers and high-income migrant workers. The results show that city public service distance has a significant nonlinear effect on the SGPHS of low-income migrant workers, but not on the SGPHS of high-income migrant workers. Figure 3c shows an inverted U-shaped relationship between city public service distance and SGPHS of low-income migrant workers, and the inflection point of this inverted U-shaped relationship is 2.708. The result reveals that city public service distance has a positive effect on the SGPHS of low-income migrant workers when the standardized index of city public service distance falls below 2.708, and a negative effect on the SGPHS of low-income migrant workers when the standardized index of city public service distance rises above 2.708. We further explore the heterogeneous effects of city public service distance on the SGPHS of migrant workers in different cities. Migrant workers are divided into an eastern city subsample and central and western city subsample according to whether their destination cities belong to eastern China.

Figure 3e,f present the heterogeneity effect of city public service distance on the SGPHS of the subgroup migrant workers in the central and western cities and the eastern cities, respectively. The results show that city public service distance has a significant nonlinear effect on the SGPHS of the migrant workers in the central and western cities, but not on the SGPHS of the migrant workers in the eastern cities. Figure 3e shows an inverted U-shaped relationship between city public service distance and SGPHS of the migrant workers in the central and western cities, and the inflection point of this inverted U-shaped relationship is 0.725. The result reveals that city public service distance has a positive effect on the SGPHS of the migrant workers in the central and western cities when the standardized index of city public service distance falls below 0.725, and a negative effect on the SGPHS of the migrant workers in those cities when the standardized index of city public service distance rises above 0.725. Figure 3f shows that the effect of city public service distance on the SGPHS of migrant workers in eastern cities is positive and statistically insignificant because there is still a more restrictive household registration system in eastern China.

### 4.6. Endogeneity Test with GPS Matching 

There may be self-selection problems between city public service distance and the SGPHS of migrant workers. For example, migrant workers with larger city public service distance tend to have a higher SGPHS, which leads to a biased effect on city public service distance and SGPHS of migrant workers. Therefore, we employ the GPS matching method by using a bootstrapping approach based on a 1000 bootstrap sample and a 95% confidence interval to test the endogeneity between city public service distance and SGPHS.

GPS matching is a generalized form of propensity score matching that allows the treatment variables to be continuous and non-normally distributed, and that mitigates bias from unobservable confounders. The GPS matching estimation of the effect of city public service distance on the SGPHS of migrant workers is according to the following three steps: (1) The propensity score is estimated by the individual characteristics, household characteristics, and city characteristics. (2) The conditional expectation of the SGPHS is estimated based on the treatment level and the propensity score. (3) The dose-response function of SGPHS on city public service distance is estimated by averaging the estimated conditional expectations [44,45,46].

**Figure 4:**
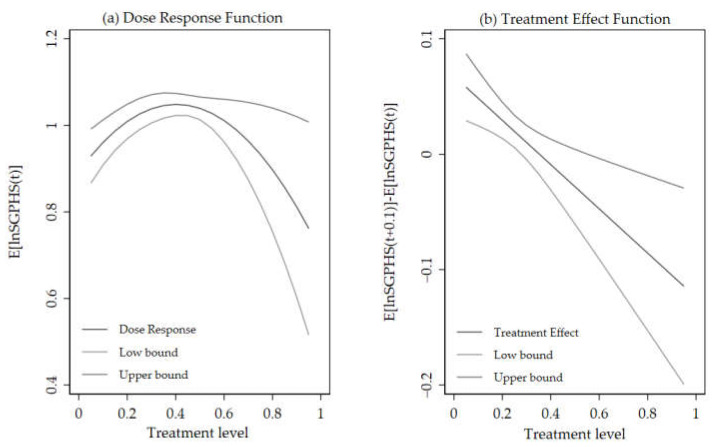
The effect of city public service distance on the SGPHS of migrant workers by generalized propensity score (GPS) matching. (**a**) City public service distance and SGPHS of migrant workers’ dose-response function. (**b**) City public service distance level and SGPHS of migrant worker’s treatment effect. Note: Low/upper bound means the lower and upper 95% confidence interval, respectively.

Figure 4 shows the treatment outcomes for GPS matching, with the SGPHS of migrant workers as the outcome variable, which reveals the association between the city public service distance level and the SGPHS of migrant workers. Figure 4a shows the average dose-response function curve, and Figure 4b reports the effect of different levels of city public service distance on the SGPHS of migrant workers, compared with the migrant workers without city public service distance. The former reveals an inverted U-shaped relationship between city public service distance and SGPHS, whereby migrant workers with the highest and lowest city public service distance tend to have a higher SGPHS than those with middle city public service distance.

Besides, we demonstrate the treatment effect of the city public service distance intensity on the SGPHS of migrant workers by calculating the difference between the SGPHS of migrant workers under different city public service distance, as shown in Figure 4b. City public service distance has a positive effect on the SGPHS of migrant workers when the intensity of city public service distance falls within the range (0, 0.35), and a negative effect on the SGPHS of migrant workers when the intensity of city public service distance falls within the range (0.35, 0.95). The impact of city public service distance on the SGPHS of migrant workers is determined by the preference reinforcement effect and public service discount effect. When the city public service distance intensity is within the range (0, 0.35), the public service distance between the destination cities and hometown is small, and then the preference reinforcement effect is bigger than the public service discount effect, thus the city public service distance has a positive effect on the SGPHS of migrant workers. When the city public service distance intensity exceeds 0.35, the public service distance between the destination city and hometown is large, and then the preference reinforcement effect is smaller than the public service discount effect, thus the city public service distance has a negative effect on the SGPHS of migrant workers.

## 5. Discussion

This is the first study to explore the public service distance of migrant workers comparing their hometown and destination cities on the SGPHS of migrant workers. This study measures SGPHS and city public service distance of migrant workers and studies the effects of city public service distance on the SGPHS of migrant workers. It provides answers to the following three questions: What is the magnitude of SGPHS for migrant workers? Does the city public service distance affect the SGPHS of migrant workers? Are there differences in the effects of city public service distance on the SGPHS for different subgroups of migrant workers? To answer these questions, this study employs a unique original dataset in 2017 including nearly 1400 migrant workers in cities and the public service statistical data of 195 cities.

### 5.1. Relationship between City Public Service Distance and the SGPHS of Migrant Workers

First, the theoretical analysis shows that the impact of city public service distance on the SGPHS of migrant workers is determined by the preference reinforcement effect and public service discount effect. The marginal effect of city public service distance is positive when the preference reinforcement effect is bigger than the public service discount effect. Besides, the marginal effect of city public service distance is negative when the preference reinforcement effect is smaller than the public service discount effect.

Second, the empirical results of the effects of city public service distance on the SGPHS of migrant workers reveal an inverted U-shaped relationship between city public service distance and SGPHS of migrant workers, whereby migrant workers with the highest and lowest city public service distance tend to have a higher SGPHS than those with middle city public service distance. The inflection point of this inverted U-shaped relationship is 2.18. When the standardized index of city public service distance falls below 2.18, the public service distance between the destination cities and hometown is relatively small, and the preference reinforcement effect is bigger than the public service discount effect, thus the city public service distance has a positive effect on the SGPHS of migrant workers. When the standardized index of city public service distance exceeds 2.18, the public service distance between the destination city and hometown is quite large, and the preference reinforcement effect is smaller than the public service discount effect, thus the city public service distance negatively affects the SGPHS of migrant workers. Besides, the endogeneity test by the generalized propensity score matching with a bootstrapping approach based on a 1000 bootstrap sample and a 95% confidence interval shows that the results are robust.

Third, the results of the effects of city public service distance on public health service accessibility and public health service provision show an inverted U-shaped relationship, respectively. The inflection point of city public service distance on public health service accessibility is higher than the provision, which reveals the greater demand of migrant workers for public health service accessibility.

Finally, in the heterogeneity analysis, (1) The results of the heterogeneity effect in terms of generation show that city public service distance has a significant nonlinear effect on the SGPHS of the second-generation migrant workers, rather than first-generation. (2) The results of the heterogeneity effect in terms of income show that city public service distance has a significant nonlinear effect on the SGPHS of low-income migrant workers, rather than high-income. (3) The results of the heterogeneity effect in terms of the region show that city public service distance has a significant nonlinear effect on the SGPHS of the migrant workers in the central and western cities, rather than eastern cities. This reveals that the effect of city public service distance on the SGPHS of migrant workers in eastern cities is positive and statistically insignificant because there is still a more restrictive household registration system in eastern China.

### 5.2. Implications

In theory, this paper contributes to the literature in three dimensions. First, this study constructs a formal evaluation framework of SGPHS, which enriches the literature related to measuring specific subjective well-being and contributes a novel perspective to the study of governance quality and social integration. The strengths of the index construction approach in this paper lie in its high transferability across regions and its flexibility in replication and application. In addition, the index construction approach can provide enlightenment for migration studies in both developed and developing countries.

Second, this study extends the theoretical framework of the cultural dimension theory to the study of public services and explains the non-linear relationship between the city public service distance and SGPHS of migrant workers in terms of the preference reinforcement effect and public service discount effect. It puts forward a clear answer as to how the city’s public service distance affects the subjective well-being of migrant workers.

Finally, this study adopts the GPS matching model to test the relationship between city public service distance and SGPHS of migrant workers, enriching the research by overcoming the ubiquitous endogeneity problem in subjective well-being.

In practice, the findings of this study have important practical guidance for improving inter-regional disparities in public services and increasing the sense of gain to public health service for migrant workers, and providing important references and insights for the formulation of health public service policies.

### 5.3. Limitations

There are some limitations to this paper. Firstly, the survey was conducted in 2017 in six representative cities of Beijing, Shanghai, Guangzhou, Zhengzhou, Wuhan, and Nanchang in China, and the sample size is 1394. However, due to the relatively restrictive household registration system in Beijing, Shanghai, and Guangzhou, migrant workers in these cities are more likely to be excluded from the urban health public service system [6,47]. As a result, the SGPHS of the respondents in these cities is relatively low, which leads to the underestimation of the effect of city public service distance on the SGPHS of migrant workers. Therefore, a stratified, multi-stage, and scale-oriented PPS sampling method in more cities and with a larger sample size is needed to facilitate empirical analysis and obtain more accurate results. Second, the attractiveness of public service is an important indicator that affects people’s use of public service, while it is not included in our questionnaire. To address the possible estimation bias caused by the omission of the attractiveness of public service, we control the city fixed effect in the empirical model. In future studies, we will consider the responses’ attitudes toward the attractiveness of public service both in the destination cities and hometowns [48], and enrich the data by adding relevant survey questions to the questionnaire. This can be achieved through further systematic research in this field.

## 6. Conclusions and Policy Implications

The findings of this study are: (1) the magnitude of SGPHS of Chinese migrant workers is at a moderate level and there are spatial differences between cities. Migrant workers in eastern regions have a higher SGPHS than those in central and western regions. (2) There is an inverted U-shaped relationship between city public service distance and SGPHS of migrant workers, and the inflection point of this inverted U-shaped relationship is 2.18, which is determined by the preference reinforcement effect and public service discount effect. Besides, the endogeneity test by the generalized propensity score matching shows that the results are robust. (3) The city public service distance has a significant inverted U-shaped effect on the public health service accessibility and public health service provision of migrant workers. The inflection point of city public service distance on public health service accessibility is higher than the provision, which reveals the greater demand of migrant workers for public health service accessibility. (4) The city public service distance has a significant inverted U-shaped effect on second-generation, low-income migrant workers, and migrant workers in central and western regions.

The empirical findings suggest thoughts and guidance on how to increase the health and well-being of migrant workers. Therefore, four policy implications may be proposed in this study. First, the reform of the equalization of public health services should concentrate on the increase of the SGPHS for migrant workers. The availability, approachability, accommodability, affordability, and acceptability of public health services for migrant workers should be increased to ensure that migrant workers have equal public health service welfare as urban *hukou* residents. Second, cities should give top priority to increasing the attractiveness of public health services, and attention should be paid to the preferences of migrant workers with large public service distances to provide them with appropriate public health services and enhance their SGPHS. Third, policymakers in eastern China should give high priority to increasing the public health service accessibility for migrant workers; liberalizing the restrictions on the public health service provision to migrant workers, and promoting the full coverage of the medical insurance system for public health. In contrast, policymakers in central and western China should increase public health spending to increase the welfare of migrant workers by adding the provision of medical and public health services. Finally, the interest demands for public health services of different subgroups of migrant workers should be considered. The coverage of public health services to first-generation migrant workers should be actively promoted, and the social integration of the second-generation migrant workers should be enhanced by promoting free movement of labour and reform of the household registration system.

## Figures and Tables

**Table 1:** The index system of the sense of gain to public health service (SGPHS) for migrant workers.

Indicators (8)	Description	Cronbach’s Alpha Reliability Coefficient
Public service accessibility		0.868
Availability	The various public resources or public services in the destination cities universally and equally benefit migrant workers.	
Approachability	The layout of public resources or public services in cities is spatially convenient, and the relatively concentrated needs of migrant workers in the urban fringe are fully considered.	
Accommodability	Service public facilities fully consider the occupational characteristics and rhythms of life of migrant workers.	
Affordability	The match between the price of fee-based public services in the destination cities and the ability to pay for the migrant workers.	
Acceptability	Whether the public service providers and their service attributes are compatible with the gender, culture, ethnicity, and other characteristics of migrant workers.	
Public health service provision		0.819
Public health service	The provision of various health services, including resident health record services, health education services, vaccination, prevention and control of infectious diseases, and medical insurance.	
Medical service	The provision of various medical services, including hospitalization, family planning services, and treatment of pneumoconiosis.	
Community management services	The provision of various city and community management services, including population registration and household registration services, food and drug safety, social security, and social conflict resolution.	

Note: All the indicators are collected at the migrant worker individual level and from the migrant worker thematic survey data in 2017. All the indicators are ordinal variables (1 = low, 3 = general, 5 = high). The Cronbach’s alpha reliability coefficient of each dimension is obtained by the reliability test.

**Table 2:** Index system of city public service distance (CPSD).

Dimensions (5)	Indicator (15)	Unit
Medical and health care (3)	Medical institutions’ supply per 10,000 people	a
	Hospital beds supply per 10,000 people	a
	Hospital doctors’ supply per 10,000 people	a
Education service (3)	Regular primary school pupil-teacher ratio	%
	Regular secondary school pupil-teacher ratio	%
	Financial expenditure on education per 10,000 people	Yuan
Culture service (2)	Books’ supply per capita in public libraries	a
	TV programs’ penetration rate	%
Social security (3)	Pension insurance penetration rate	%
	Basic medical insurance penetration rate	%
	Unemployment insurance penetration rate	%
Infrastructure (4)	Mobile phone penetration rate	%
	Total gas (coal gas, natural gas) penetration rate	%
	Public vehicles’ supply per 10,000 people	a
	The green space penetration rate in built-up areas	%

Note: All the indicators are collected at the city level and from the China Statistical Yearbook.

**Table 3:** Statistical summary of the variables.

Variable	Description	Symbol	Mean	SD	Min	Max
Dependent variables						
Sense of gain to public health service	Sense of gain to public health service score	SGPHS	2.96	0.79	1.00	4.98
Public health service accessibility	Public health service accessibility score	PHSA	3.03	0.81	1.00	5.00
Public health service provision	Public health service provision score	PHSP	2.89	0.88	0.99	4.95
Independent variables						
City public service distance	City public service distance score	CPSD	5.92	4.27	0	72.47
Individual characteristics						
Male	1 = male, 0 = female	male	0.53	0.49	0.00	1
Age	year	age	34.02	10.56	15	71
Education status						
Junior middle school	1 = yes, 0 = no	junedu	0.43	0.48	0	1
Senior high school	1 = yes, 0 = no	senedu	0.28	0.45	0	1
Junior college and above	1 = yes, 0 = no	college	0.15	0.42	0	1
Married	1 = yes, 0 = no	married	0.57	0.47	0	1
Individual income	Yuan	income	4.92	3.66	1	20.00
Household characteristics						
Household scale	Household scale	hhsize	4.57	1.89	1	23
City characteristics						
Eastern China	1 = yes, 0 = no	eastcity	0.62	0.49	0	1
Average wage	Yuan	average	9.57	2.48	6.12	12.28
Inter-city characteristic						
City geodesic distance	(in 100) kilometers	CGD	4.92	4.22	0	30.64

**Table 4:** Effects of City Public Service Distance on the SGPHS of migrant workers.

	Model (1)	Model (2)	Model (3)	Model (4)
	Coefficient (SE)	Coefficient (SE)	Coefficient (SE)	Coefficient (SE)
CPSD (sd)	0.094 ***	0.094 ***	0.091 ***	0.109 **
	(0.034)	(0.034)	(0.034)	(0.045)
CPSD (sd)-squared	−0.024 ***	−0.023 ***	−0.023 ***	−0.025 ***
	(0.006)	(0.006)	(0.006)	(0.007)
male		−0.014	−0.017	−0.049
		(0.057)	(0.057)	(0.057)
age		−0.069 ***	−0.070 ***	−0.071 ***
		(0.016)	(0.016)	(0.016)
age-squared		0.001 ***	0.001 ***	0.001 ***
		(0.000)	(0.000)	(0.000)
junedu		0.098	0.093	0.097
		(0.093)	(0.093)	(0.091)
senedu		0.175 *	0.169*	0.175 *
		(0.099)	(0.099)	(0.097)
college		0.181 *	0.169	0.195 *
		(0.108)	(0.108)	(0.106)
married		0.017	0.022	0.025
		(0.071)	(0.072)	(0.071)
lnincome		0.127 ***	0.129 ***	0.031
		(0.048)	(0.049)	(0.050)
lnhhsize			−0.017	−0.023
			(0.069)	(0.068)
eastcity				0.377 ***
				(0.056)
lnwage				−0.129
				(0.163)
CGD(sd)	0.034	0.038	0.040	0.040
	(0.029)	(0.028)	(0.029)	(0.029)
Constant	0.024	0.814 ***	0.852 ***	1.092 **
	(0.027)	(0.291)	(0.306)	(0.507)
Observations	1.394	1.393	1.382	1.382
R-squared	0.216	0.239	0.239	0.268

Note: The variables of CPSD (sd) and CGD (sd) are the CPSD and CGD taken in a standardized form. Standard errors are displayed in the parentheses below all coefficients. ***, **, and * denote the 1%, 5%, and 10% significance levels, respectively.

**Table 5:** Results of CPSD on public health service accessibility and provision.

	Model (5)	Model (6)
	Coefficient (SE)	Coefficient (SE)
CPSD (sd)	0.090 * (0.046)	0.114 *** (0.044)
CPSD (sd) -squared	−0.019 *** (0.005)	−0.027 *** (0.008)
male	−0.041 (0.057)	−0.049 (0.056)
age	−0.055 *** (0.016)	−0.077 *** (0.017)
age-squared	0.001 *** (0.000)	0.001 *** (0.000)
junedu	0.110 (0.093)	0.073 (0.089)
senedu	0.170 * (0.099)	0.158 * (0.095)
college	0.226 ** (0.108)	0.144 (0.104)
married	0.056 (0.071)	−0.006 (0.071)
lnincome	0.016 (0.050)	0.042 (0.049)
lnhhsize	−0.045 (0.066)	0.001 (0.069)
eastcity	0.383 *** (0.056)	0.326 *** (0.057)
lnwage	−0.184 (0.166)	−0.062 (0.160)
CGD (sd)	0.044 (0.029)	0.033 (0.030)
Constant	0.983 * (0.514)	1.058 ** (0.505)
Observations	1.382	1.382
R-squared	0.256	0.267

Note: The variables of CPSD (sd) and CGD (sd) are the CPSD and CGD taken in a standardized form. Standard errors are displayed in the parentheses below all coefficients. ***, **, and * denote the 1%, 5%, and 10% significance levels, respectively.

## Data Availability

Data used in this research were provided by the key research base of humanities and social science in the Ministry of Education of China. The datasets produced and analyzed for this study are available from the corresponding author on reasonable request.
